# Has telemedicine come to fruition? Parents’ and pediatricians’ perceptions and preferences regarding telemedicine

**DOI:** 10.1038/s41390-024-03172-w

**Published:** 2024-03-30

**Authors:** Nadia M. Bajwa, Noelle Junod Perron, Olivia Braillard, Sophia Achab, Patricia Hudelson, Melissa Dominicé Dao, Robin Lüchinger, Sanae Mazouri-Karker

**Affiliations:** 1 Department of General Pediatrics at the Children’s Hospital, Geneva University Hospitals, Geneva, Switzerland; 2 Unit of Development and Research in Medical Education, Faculty of Medicine, University of Geneva, Geneva, Switzerland; 3 Medical Directorate, Geneva University Hospitals, Geneva, Switzerland; 4 Primary Care Division, Department of Primary Care Medicine, Geneva University Hospitals, Geneva, Switzerland; 5 Clinical and Sociological Research Unit, WHO Collaborating Centre for Training and Research in Mental Health, Geneva, Switzerland; 6 Treatment Centre ReConnecte, Department of Psychiatry, University Hospitals of Geneva, Geneva, Switzerland; 7 E-health and Telemedicine Division, Geneva University Hospitals, Geneva, Switzerland

## Abstract

**Background:**

Telemedicine has increasingly become a viable option for patient care and may increase access to care. The aim of our study was to evaluate both parent and pediatrician perceptions, preferences, and acceptability regarding the use of different telemedicine modalities.

**Methods:**

We conducted a cross-sectional survey of both parents and pediatricians in Geneva, Switzerland in 2021. The questionnaire focused on digital literacy, preferences, acceptability, advantages, and disadvantages regarding telemedicine (phone, email, video, and instant message). Descriptive statistics and comparisons of preferences and perceptions (Pearson Chi^2^ and logistic regression) were performed.

**Results:**

Two hundred and twenty-two parents and 45 pediatricians participated. After face-to-face consultations, parents and pediatricians preferred the phone for simple medical advice, discussion of parameters, acute or chronic problems, and psychological support. Email was preferred for communication of results and prescription renewal. Main reasons for using telemedicine were avoiding travel and saving time. Disadvantages were lack of physical examination, technical problems, and unsuitability of the reason for consultation.

**Conclusions:**

Understanding the factors that influence acceptance and satisfaction with telemedicine is vital for its successful implementation. Convenience, quality of care, trust, strong pediatrician–parent relationships, technical reliability, user-friendliness, and privacy considerations play significant roles in shaping parent and pediatrician attitudes toward telemedicine.

**Impact:**

- The COVID-19 pandemic spurred the expansion of the use of telemedicine in pediatric care. Few studies have addressed parent and pediatrician perceptions and preferences regarding telemedicine.
- Both parents and pediatricians consider certain telemedicine modalities (phone, email, video, and instant message) pertinent in only specific clinical situations.
- Advantages of telemedicine outweigh disadvantages with parents and pediatricians appreciating the increased access to care, time savings, and avoiding transport. However, the lack of a physical examination remains a significant disadvantage.
- Convenience, quality of care, trust, strong pediatrician–parent relationship, technical reliability, user-friendliness, and privacy considerations play significant roles in shaping attitudes towards telemedicine.

## Introduction

During the past 10 years, the literature on the use of telemedicine in pediatrics has grown exponentially. Telemedicine encompasses the use of either synchronous (phone, video) or asynchronous (email, text message) technologies to provide healthcare at a distance. The coronavirus disease 2019 (COVID-19) pandemic transitioned telemedicine from an adjunct method of delivering care to a necessity internationally.^1^ The pandemic provided a stimulus to use telemedicine as a means of maintaining quality pediatric care by enhancing the consultation experience by maintaining quality and satisfaction, improving population health, reducing healthcare costs, and improving pediatrician work–life integration.^2,3^

Telemedicine increases access to care by allowing families to bypass transportation difficulties and time away from work, waiting times, and exposure to communicable diseases that may occur in the clinic that are sources of decreased patient satisfaction.^2,4,5^ At the same time, telemedicine may increase continuity of care by facilitating access to the child’s pediatrician. Telemedicine has also been used to increase access to subspecialists that would have otherwise been inaccessible.^4,6,7^ For clinicians, telemedicine reduces the risk of contamination and increases physician flexibility regarding work hours.^1,2^ Telemedicine may also be a more effective way of limiting patient portal questions through more direct communication compared to asynchronous communication.^2^

Downsides to telemedicine have also been reported. When using telemedicine, there are higher rates of inappropriate antibiotic prescriptions by pediatricians highlighting the need for rigorous quality control.^8^ Payers have voiced their concern that telemedicine could result in an overuse of healthcare utilization for self-limited illnesses as reported in adult studies.^9^ There exists also the hypothetical risk that a decrease in in-person visits could negatively affect vaccination rates.^2^ For those who have limited digital literacy, telemedicine may increase inequity in access to care.^10^ In addition, paying special attention to the needs of foreign language speaking and deaf patients must be considered in the implementation of telemedicine programs.^1^

Few studies have explored parent and pediatrician perceptions and preferences concerning telemedicine. One such study concerning a well-established pediatric telemedicine program in Israel demonstrated that physicians were concerned about the accuracy of their diagnoses, difficulties in establishing a relationship with new patients, feeling isolated from colleagues due to the setting of their telemedicine workplace, feeling pressured to quickly make diagnostic judgments, and technological challenges.^11^ One recent review though found that patients demonstrated high satisfaction (85%) with their telemedicine encounters.^10^ While it is clear that the COVID-19 pandemic provided a needed stimulus for telemedicine, recent studies in our setting have shown that adult patients and physicians may find telemedicine acceptable, but continue to prefer in-person visits to different telemedicine modalities.^12^

The aim of this study is to explore the preferences, perceptions, and challenges related to telemedicine as perceived by both parents and pediatricians, post-pandemic in Geneva, Switzerland.

## Methods

### Design

We administered a cross-sectional survey to both parents and pediatricians in Geneva, Switzerland between September 2021 and January 2022. Two separate surveys were administered to parents and pediatricians. Development of the survey was initially based on a preliminary literature review of patient and physician experiences with telemedicine.^13–16^ Our questionnaire was mainly inspired by one created for an adult population in a similar study.^12^ Each survey consisted of 27 items and was administered electronically. Survey items were created by the research team and consisted of participant sociodemographic characteristics, level of digital literacy, communication preferences for consultations (face to face, phone, video, email, and instant message ranked from 1 to 5), and the acceptability of different telemedicine formats for specific clinical situations. Digital literacy items were focused on digital usage and were inspired by a pre-existing digital literacy scale.^17^ The clinical situations consisted of nine common healthcare situations experienced by patients and treated by pediatricians: information transmission of test results (receiving or providing), medical advice (receiving or providing), discussion of patient monitored observations (weight, blood sugar, etc.), monitoring of a chronic health problem, follow-up of an acute health problem, support for mental health and psychosocial issues (receiving or providing), request for referral to a specialist, request for a school or work excuse, and renewal of a prescription. Participants were asked to indicate their agreement (yes/no) with each format of telemedicine.

Specific questions developed for pediatricians included barriers regarding the use of phone and video (open-ended responses). In addition, parents were questioned about their perception of confidentiality and data security of phone and video consultations (Likert scale from 1 totally disagree to 5 totally agree). A pediatrician in the research group (N.M.B.) verified that the survey items and the clinical situations were appropriate for a pediatric setting. Questionnaires were then pilot tested with 10 patients and 10 physicians. Changes were made to ensure that the content of the survey represented the experiences of parents and pediatricians, as well as for clarity and comprehension, resulting in seven iterations before finalizing the survey. The patient questionnaire was translated from French into English, Portuguese, and Spanish (the three most common foreign languages spoken in Geneva) by native speakers. The translated questionnaires were then back translated to verify congruence. The survey was imported into the online survey software Qualtrics.^18^ The survey items can be found in Appendices 1 and 2.

### Ethical considerations

As this study did not collect personal health information, the cantonal Ethical Committee for the Canton of Geneva granted an exemption from ethical review (article 2 of the Swiss Federal Act on Research involving Human Beings). Nonetheless, the survey was anonymous, and participants completed written informed consent before the administration of the survey.

### Participant recruitment

Recruitment sites included three walk-in clinics (two private and one public hospital) and one primary care medical center. Research assistants invited all French, English, Spanish, and Portuguese speaking parents over 18 years of age to complete the online survey. Parents were randomly recruited in the waiting rooms of the study sites. Parents were free to complete the survey on a supplied tablet or with their personal phone via a QR code. Parents received a 10 Swiss franc voucher for their participation.

Primary care pediatricians were recruited by obtaining email addresses from the Geneva Medical Association. Email invitations were sent to all pediatricians working in outpatient settings. Reminder emails were sent at both 2 and 4 weeks after the initial invitation.

### Analysis

Descriptive statistics were conducted for both parent and pediatrician preferences and opinions concerning the acceptability of different telemedicine modalities. Differences between the two groups were analyzed using Chi Square. Differences in parents’ perceptions of confidentiality regarding the use of phone vs video for the consultation were analyzed with the McNemar test. Relationships between parent characteristics and advantages and disadvantages of telemedicine were analyzed using logistic regression with multivariate analysis. For this analysis, advantages and disadvantages were categorized as timesaving, facilitated access, lack of physical contact, and technical aspects. A *P* value ≤ 0.05 was considered statistically significant. All analyses were conducted with Stata Statistical Software, Release 15.^19^

## Results

### Participants

Two hundred and twenty-two parents completed the questionnaire. The parental response rate was 60%; reasons for refusal were not documented. The average age of their child was 7 years (5.6 SD). Forty-five pediatricians participated (38% response rate). The average age was 45 years (11.7 SD). Descriptive demographic statistics of the participants can be found in Table 1. A summary of both parents’ and pediatricians’ digital literacy can be found in Table 2.

**Table 1:** Participant characteristics.

Sociodemographic data	Parents *n* = 222 *n* (%)	Sociodemographic data	Pediatricians *n* = 45 *n* (%)
Gender of the child		Gender	
Boy	114 (51%)	Men	10 (22%)
Girl	108 (48%)	Women	35 (78%)
Primary language		Practice location	
French	182 (82%)	Private practice	19 (42%)
English	22 (10%)	Public institution	26 (58%)
Portuguese	16 (7%)		
Spanish	2 (1%)		
Working time of parents		Working time of pediatricians	
Full time	104 (47%)	Full time	23 (51%)
Part time	67 (30%)	Part time	22 (49%)
Unemployed	51 (23%)		
Perceived health status of children		Years of work experience	
Excellent	73 (33%)	<5 years	5 (11%)
Very good	78 (35%)	5–10 years	5 (11%)
Good	53 (24%)	>10 years	35 (78%)
Fair	13 (6%)		
Poor	5 (2%)		
Established care with a pediatrician	211 (95%)		
Duration of pediatrician-patient relationship			
<2 years	76 (34%)		
2–5 years	53 (24%)		
>5 years	93 (42%)		
Frequency of medical consultation			
1×/year	30 (13.5%)		
2×/year	51 (23%)		
3–4×/year	71 (32%)		
5–12×/year	57 (26%)		
>12×/year	13 (6%)

**Table 2:** Participants’ access to and use of connected devices.

Digital use data	Parents	Digital use data	Pediatricians
Internet access		Internet access	
Yes	220 (99.1%)	Yes	45 (100%)
No	2 (.9%)	No	0 (0%)
Frequency of internet use			
Everyday	212 (95.5%)		45 (100%)
A few times a week	7 (3.2%)		0 (0%)
A few times a month	2(.9%)		0 (0%)
Less than once/month	1(.45%)		0 (0%)
Connected devices		Connected devices	
Computer	181 (81.5%)	Computer	45 (100%)
Smart phone	215 (96.9%)	Smart phone	45 (100%)
Tablet	125 (56.3%)	Tablet	29 (64.4%)
Other	8 (3.6%)	Other	0 (0%)
Usage of connected devices		Usage of connected devices	
Phone calls	197 (88.7%)	Phone calls	43 (95.6%)
Video calls	159 (71.6%)	Video calls	30 (66.7%)
Email	202 (91.0%)	Email	45 (100%)
Instant message	208 (93.7%)	Instant message	41 (91.1%)
Work	139 (62.6%)	Work	41 (91.1%)
Information seeking	167 (75.2%)	Information seeking	45 (100%)
Gaming	93 (41.9%)	Gaming	27 (60.0%)
Consultation format previously used		Utilization of an electronic medical record	41 (91.1%)
Phone	117 (52.7%)		
Email	61 (27.5%)		
Video	2 (.9%)		
Instant message	18 (8.1%)		
None	31 (14.0%)		

### Preferences for telemedicine

**Fig. 1:**
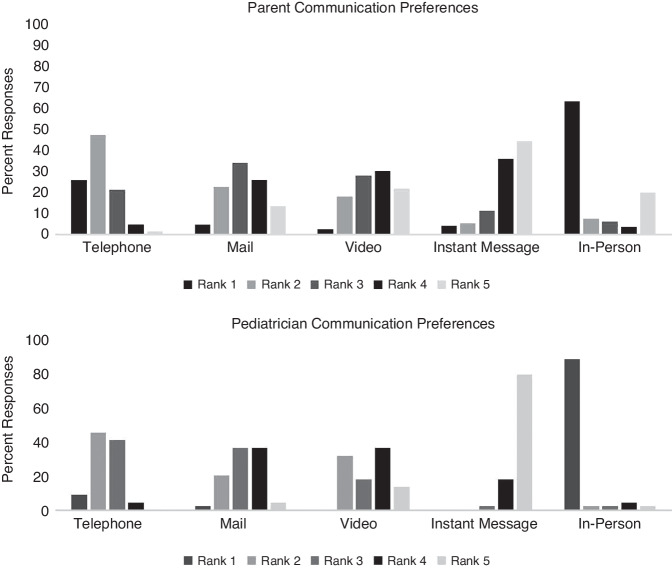
Parent and pediatrician communication preference rankings.

Pediatricians and parents ranked their preferences for types of consultation (in-person, phone, mail video, and SMS text message) differently; see Fig. 1. Among pediatricians, in-person obtained the highest rank (89%), parent preferences were similar but to a lesser extent (63.3%). Parents selected phone a quarter of the time (25.7%) while phone was chosen only by 9.1% of pediatricians. The first ranked option was significantly different between pediatricians and parents (*P* < 0.001, Chi^2^ test).

**Fig. 2:**
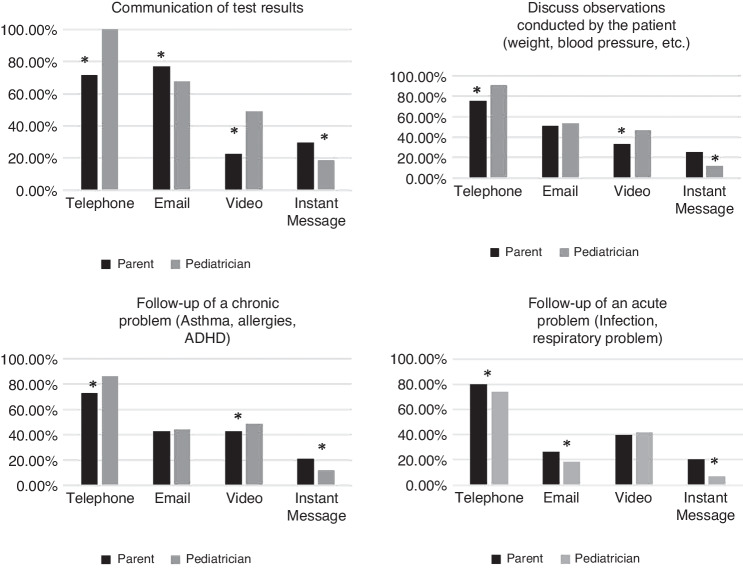

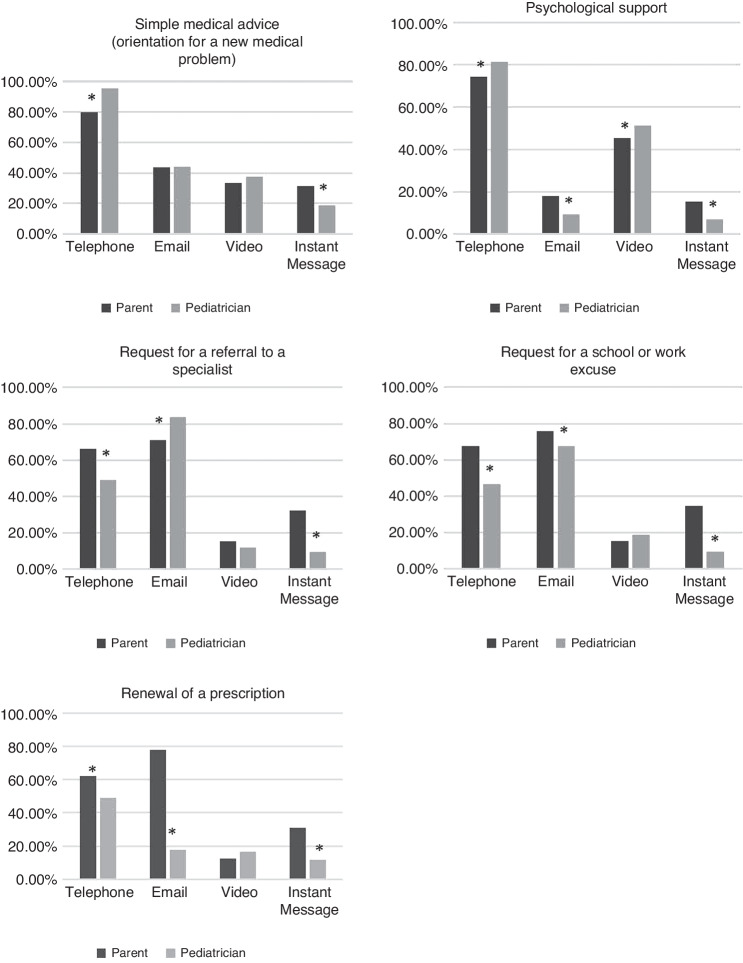
Acceptability of different telemedicine modalities in relation to different clinical situations. Note. *Indicates a significant Pearson Chi^2^ value of *P* ≤ 0.05.

Parents and pediatricians were also questioned on acceptable modalities of telemedicine in relation to specific clinical situations (Fig. 2). Significant differences in rates of acceptability for the different types of clinical situations were found between parents and pediatricians. However, in both groups, the phone was the preferred modality for most clinical situations except for administrative requests such as referrals, excuses, or prescriptions where email was found to be the most acceptable modality.

When questioned about their opinions on the confidentiality and utility of video versus phone consultations, parents found that both options ensured confidentiality (68.5% for video and 76% for phone; *P* = 0.057). However, parents felt that it was easier to communicate their needs through the phone than the video (70% phone vs. 60% video; *P* = 0.029). Both modalities were found to allow pediatricians to comprehend the problem at hand (52% video and 52% phone). However, in general, parents rated the quality of video and phone consultations comparable to in-person consultations at a lower rate (25% for video and 26% for phone).

### Advantages and disadvantages of telemedicine

Parents and pediatricians displayed differences in perceptions of advantages and disadvantages of telemedicine; see Table 3. Parents cited the following top five advantages to using telemedicine: no need for transport (67%), time savings (59%), faster access to medical care or advice (44%), avoids having to take time off work (37%), and avoids having to visit an emergency center or an alternative pediatrician (36%). The most frequently cited disadvantages for parents were the following: lack of a physical examination (68%), medical situation is not always amenable to telemedicine (44%), technical problems which may occur (38%), contact with the pediatrician being less warm and less friendly (27%), and not allowing for frequent health monitoring (22%).

**Table 3:** Parents’ and pediatricians’ perceived advantages and disadvantages of telemedicine.

	Parents	Pediatricians
	*N* (%)	*N* (%)
Advantages		
*Gain of time*		
Saves me a trip	149 (67.1)	32 (71.1)*
Saves me time	131 (59.0)	11 (24.4)*
Shorter consultation	40 (18.0)	9 (20.0)*
Saves childcare arrangement	56 (25.2)	9 (20.0)*
Less time off work	83 (37.4)	9 (20.0)*
Doctor more punctual/more time flexibility	13 (5.9)	45 (11.4)*
Facilitated access to treatment		
Faster access	98 (44.1)	15 (33.3)*
Follow-up while abroad	40 (18.0)	16 (35.6)*
More frequent follow-up	11 (5.0)	9 (20.0)*
Avoid emergency center consultation	81 (36.5)	15 (33.3)*
Other reasons		
Less expensive consultations	39 (17.8)	6 (13.3)*
Easier for care giver participation	25 (11.3)	11 (24.4)*
Talks about more things	7 (3.2)	11 (24.4)*
Feels less anxious	13 (5.9)	9 (20.0)*
No benefit	21 (9.5)	2 (4.4)*
*Disadvantages*		
Deficient physical contact/communication	150 (67.6)	39 (86.7)*
No physical exam	29 (13.1)	13 (28.9)*
Less quality communication	60 (27.0)	24 (53.3)*
Less warm/friendly contact	27 (12.2)	2 (4.4)*
Less active patient participation	6 (2.7)	0 (0%)
Too intrusive on my privacy		
Technical aspects		
Technical problems (image, sound)	84 (37.8)	27 (60.0)*
Requires specific software	27 (12.2)	2 (4.4)*
Confidentiality/security		
Lack of confidentiality	30 (13.5)	15 (33.3)*
Lack of security	27 (12.2)	11 (24.4)*
Does not lend itself to the medical situation	98 (44.1)	34 (75.6)*
Other reasons		
Fewer problems addressed	22 (9.9)	3 (6.7)*
Does not allow for frequent health monitoring	49 (22.1)	2 (4.4)*
Call at an inconvenient time	28 (12.6)	2 (4.4)*
No disadvantages	16 (7.2)	0 (0%)

*P value ≤ 0.05, Pearson Chi^2^.

The most noted advantages to telemedicine in the eyes of pediatricians were decreased patient transport time (71%), ability to follow patients who were abroad (36%), ability to deliver a quicker consultation or response time (33%), and that patients did not need to consult another pediatrician or center (33%). The most striking disadvantages were that telemedicine may not be adapted to the type of clinical situation (76%), the possibility of technical problems (60%), and the impossibility to guarantee the confidentiality of the consultation (33%).

The relationship between telemedicine advantages and disadvantages and parent characteristics such as gender, working time, and educational level were explored with logistic regression. The only significant relationship found was that of time saving by parents who have completed vocational training, odds ratio (OR) = 11.11 (95% confidence interval (CI) 1.59–77.82), or university education OR = 10.26 (95% CI 1.69–62.42). There were no significant characteristics related to increased access to patient care nor to disadvantages such as the lack of physical contact or changes in communication style.

## Discussion

In our study, telemedicine was shown to be an acceptable method of patient consultation. We were able to demonstrate that parents and pediatricians have different preferences and levels of acceptability for different telemedicine modalities. Both parents and pediatricians agreed with the use of phone and email for certain clinical situations and administrative tasks, while video and SMS text message fell behind in terms of preference and acceptability. These findings are similar to adult patient and physician studies where email was more largely accepted for simple medical advice and provision of documents in comparison to SMS text message.^20–22^ For both parents and physicians, video was an acceptable telemedicine modality when psychological support is needed. Literature has shown that integration of parents in the teleconsultations and having choices such as phone or video seemed to be key factors for adoption of telepsychiatry follow-up for teenagers.^23^

Similar to the literature, not all pediatricians and patients in our study felt reassured regarding the confidentiality of the consultation.^24^ Our previous study showed that adults and their physicians expressed more trust in phone than video.^12^ This differs from the current study where parents found that both options (phone and video) ensured confidentiality in contrast to pediatricians who were more concerned about their ability to maintain the confidentiality of the consultation. However, parents felt that neither video nor phone provided the same quality of care as an in-person consultation. This is contrary to the literature where remote consultations by phone or video were considered as an effective alternative to face-to-face consultations in terms of patient satisfaction and costs in primary care and mental health services.^25^

Advantages of telemedicine conferred by parents in this study are also similar to those described in the literature with time gains in terms of transport and convenience as major motivators to using telemedicine.^26,27^ Pediatricians also described similar advantages in noting that the gain of time and convenience helped to ensure continuity of care when the patient did not consult elsewhere. Similar results were found in our study of an adult population where gain of time and facilitated access was highly rated for both patients and physicians, with patients giving more importance to gain of time.^12^

Disadvantages in our study mirrored those found in our study with an adult population such as absence of physical contact, lower quality communication, technical problems, confidentiality issues, and inappropriateness of the clinical situation.^12^ Specifically, parents were wary of the limits of telemedicine when it came to the lack of the physical examination and the potential lack of a personal relationship with the pediatrician. Both parents and pediatricians worried about potential technical problems that could hinder the consultation and that the reason for the visit would not be amenable to using telemedicine. These disadvantages have led to concerns regarding patient safety in the literature. The risk for diagnostic error is increased as the pediatrician has limited objective information to rely upon, the inability to perform specific diagnostic maneuvers, and the lack of contextual information from full visualization of the patient.^28,29^ These limitations in communication may also result in medication errors as visual cues and written education used to verify understanding are more difficult to employ.^28^

Despite these noted disadvantages, it is possible to increase the acceptability of telemedicine by implementing training for pediatricians. Pediatricians need to pay special attention to patient familiarity and digital literacy with telemedicine modalities.^4^ Telemedicine requires a subset of communication skills that pediatricians should familiarize themselves with in order to improve their acceptance of and comfort with virtual consultations.^4,30,31^ For example, pediatricians should ensure a secure environment, address confidentiality, and emphasize skills used to develop the relationship and structure of the consultation.^32,33^ Training pediatricians to specifically address these aspects of the consultation may help parents to put aside these concerns.^34^ While the lack of a physical examination was noted as a major disadvantage of telemedicine, it is possible to train pediatricians in techniques that simulate the physical examination and allow pediatricians to collect relevant clinical information. Specific telemedicine curricula addressing these issues have been shown to improve both patient and physician satisfaction with the consultation.^30^

Additional opportunities exist to improve the reputation of telemedicine.

For example, platforms that provide telemedicine need to be simple and user friendly so that difficulties related to technical problems are infrequent.^31^ Both parents and pediatricians also expressed their concerns about the adequation of the clinical complaint with a telemedicine consultation. Practices should be explicit about what types of complaints and services are amenable to telemedicine.^5^ This would allow families to approach the consultation with reasonable expectations concerning the outcome. Ultimately, meeting patient expectations is the focus of patient-centered care. Studies have shown that despite the limitations in communication, telemedicine can enhance patient-centered care if physicians make efforts to personalize care by guiding the patient and their family through the consultation, by offering appropriate health information resources, by ensuring attentive listening, and by conveying respect for the patient.^35,36^ Future studies may seek to compare consultations for specific clinical situations using either in-person, phone, or video consultation. Specifically, these studies may address new patients vs established patients, the effects on shared decision making, impact on treatment adherence, ethical issues, impact of digital divide and healthcare cost coverage on access to telemedicine, and outcomes related to quality of life.

The adoption of telemedicine has been shown to be influenced by social determinants of health such as socioeconomic status, geographic location, education level, insurance status, digital literacy, language barriers, and race.^37–39^ Our participant sample demonstrated high levels of digital literacy with the vast majority of participants having internet access and using electronic devices for multiple purposes daily. This may be due to the younger age demographic of the parents in our sample who are more likely to be digital natives. Concerning insurance status, Switzerland has a universal health coverage model where patients who cannot afford health insurance are subsidized by the government. This ensures that access to healthcare is equitable. The educational level of our participants was not found to significantly influence the advantages or disadvantages associated with telemedicine except for time-savings. Our study did not specifically address social health determinants such as socioeconomic status, geographic location, language barriers, or race, however, future studies in other health care contexts should address these social health determinants when investigating the effectiveness of telemedicine.

### Limitations

Limitations to our study include the sampling from a specific urban cantonal region in Switzerland and the modest response rate. Although, when compared to the literature on physician survey response rates our rates are typical.^40^ Future studies that question all users of an existing telemedicine platform may confirm or give new insights into the findings already established in our study. Specifically, seeking responses from more rural areas may expand the generalizability of our results. There is the possibility that non-responders to our study may have been pediatricians or parents with low levels of digital literacy. Follow-up studies should look to include these groups specifically to identify those barriers that need to be overcome in the implementation of telemedicine. The recruitment sites consisted primarily of urgent care centers located in urban locations and this may have influenced the type of participants in our study. While public transport in Geneva, Switzerland is well developed and access to care is usually not limited by distance, ongoing studies should seek out to include patients and parents in existing primary care practices in diverse locations. Finally, our study did not address the financial aspects of telemedicine and the potential cost-savings of engaging telemedicine. These aspects should be included in future studies so as to gain insight into this cost-benefit ratio.

## Conclusion

Understanding the factors that influence both parental and pediatrician acceptance of and satisfaction with telemedicine is vital for its successful implementation. Convenience, quality of care, trust, strong pediatrician–parent relationships, technical reliability, user-friendliness, and privacy considerations play significant roles in shaping parental attitudes towards telemedicine. Pediatrician uptake of telemedicine may be influenced by training related to telemedicine specific communication and physical examination skills to improve their self-efficacy during teleconsultations.^41^ By addressing these factors, healthcare providers and policymakers can enhance the acceptance and satisfaction of both parents and pediatricians, promoting the wider adoption of telemedicine in pediatric care.

## Supplementary information

Supplementary Files Appendix 1 and 2 Telmed Ped

## Data Availability

The datasets generated during and/or analyzed during the current study are available in Yareta: 10.26037/yareta:dpnmoplvjfginfga4djnkskefu
