# Supplementary material for: Has telemedicine come to fruition? Parents’ and pediatricians’ perceptions and preferences regarding telemedicine
Source: Pediatr Res. 2024 Mar 30;96(5):1332–9. doi: 10.1038/s41390-024-03172-w (PMC11522004; doi:10.1038/s41390-024-03172-w)
Supplement: Supplementary file 1 — Supplementary Files Appendix 1 and 2 Telmed Ped [file 41390_2024_3172_MOESM1_ESM.pdf]

Appendix 1: Telemedicine: Pediatrician Survey

Start of Block: Default question block

Q1
You are invited to participate in a survey conducted by a team from the University Hospitals of Geneva (HUG) and the University of Geneva. It has been submitted to the Commission Cantonale d'Ethique de la Recherche en Suisse (exemption granted).

The purpose of this study is to evaluate your perceptions and preferences regarding the modes of communication in telemedicine, i.e., for health care or advice delivered by telephone, video, email or instant messaging. The results of this research will help to propose recommendations for best practices.

Your participation consists of completing a questionnaire that should only take 10 minutes. You are free to accept or decline to participate in the survey. You decision and withdraw from the project at any time without having to justify your decision. project without having to justify yourself.

The data collected for research purposes are anonymous. The results may be used in scientific publications. The confidentiality of the data will be ensured, and your name or any your name or any information that could identify you will not appear anywhere. All persons involved in the study are bound by confidentiality. confidentiality.

You You may contact the principal investigator of the survey at any time sanae.mazouri@hcuge.ch / +41795530154

By agreeing to participate in this study, you consent to the use of your data for research purposes.

- ○ I wish to participate in the study. (1)
- ○ I do not wish to participate in the study. (2)

Skip To: End of Survey If you are invited to participate in a survey conducted by a team from the University Hospitals of... = I do not wish to participate in the survey.

End of Block: Default question block

Start of Block: Block 1

*

Q2 What is your year of birth (e.g. 1987)

____

Q3 What is your gender?

- Male (1)
- Female (2)
- Other (3)

Q4 What is your medical specialty?

- ☐ Anesthesia (1)
- ☐ Angiology (8)
- ☐ Allergology-immunology (9)
- ☐ Cardiology (10)
- ☐ Surgery (specify) (11) ________________
- ☐ Dermatology (2)
- ☐ Endocrinology / diabetology (3)
- ☐ Gastroenterology (4)
- ☐ Medical genetics (5)
- ☐ Gyneco-obstetrics (7)
- ☐ Hematology (12)
- ☐ Infectiology (13)
- ☐ Occupational medicine (14)
- ☐ General Internal Medicine (15)
- ☐ Physical medicine and rehabilitation (16)
- ☐ Tropical medicine (17)

- [ ] Nephrology (18)
- [ ] Neurology (19)
- [ ] Oncology (20)
- [ ] Ophthalmology (21)
- [ ] ENT (22)
- [ ] Pediatrics (23)
- [ ] Pharmacology (24)
- [ ] Pneumology (25)
- [ ] Adult psychiatry (26)
- [ ] Child and adolescent psychiatry (27)
- [ ] Radiology (28)
- [ ] Radio-oncology (29)
- [ ] Other (6) ____

Q5 What is your primary township of practice?

- AG (1)
- AI (2)
- AR (3)
- BE (4)
- BL (5)
- BS (6)
- EN (7)
- GE (8)
- GL (9)
- GR (10)
- JU (11)
- LU (12)
- NE (13)
- NW (14)
- OW (15)
- SG (16)
- SH (17)
- SO (18)
- SZ (19)
- TG (20)
- IT (21)

- UR (22)
- VD (23)
- VS (24)
- ZH (25)
- ZG (26)

Q6 What is your primary place of practice?

- Urban (1)
- Semi-urban (2)
- Rural (3)

Q7 What is your primary facility type?

- Individual (1)
- 2 - 4 physicians (2)
- Medical Center (3)
- Hospital / Institution (4)

Q8 What is your activity rate?

	0	10	20	30	40	50	60	70	80	90	100
Activity rate in percent ()											

Q9 How many years of experience have you had since obtaining your federal degree or equivalent?

- < 5 years (1)
- 5-10 years (2)
- > 10 years (3)

End of Block: Block 1

Start of Block: Block 2

Q10 Do you have access to the Internet at home?

- Yes (1)
- No (2)
- I don't know (3)

Q11 What connected tools do you have?

- Computer (1)
- Smartphone (2)
- Tablet (3)
- Other (4) ____
- None (5)

Display This Question:

If What connected tools do you have? = None

Q12 How often do you use these connected tools?

- ○ Every day (1)
- ○ A few times a week (2)
- ○ A few times a month (3)
- ○ Less than once a month (4)
- ○ Never (5)

Display This Question:

If What connected tools do you have? = None

Q13 How do you use your connected tools?

- ☐ Making calls (1)
- ☐ Make video calls (2)
- ☐ Send - receive emails (3)
- ☐ Send -receive instant messages (4)
- ☐ Working (5)
- ☐ Search for information (7)
- ☐ Gaming / social networks / online shopping (6)

Q14 Does your practice/office have computer software for managing patient records?

- Yes (1)
- No (2)
- I don't know (3)

End of Block: Block 2

Start of Block: Block 3

Q15 In your interactions with your patients, please indicate the means of communication other than traditional face-to-face that you feel are acceptable for the following situations (multiple choices possible):

	Telephone (1)	Mail (2)	Video (3)	Instant messaging (4)
Communication of test results (laboratory, radiology) (1)	☐	☐	☐	☐
Discussion of self-measured values at home (blood pressure, sugar level, pain intensity, ...) (2)	☐	☐	☐	☐
Follow-up for a chronic disease/problem (high blood pressure, diabetes, osteoarthritis, etc.) (3)	☐	☐	☐	☐
Follow-up of an urgent illness / problem (urinary infection, cold, gastroenteritis, back blockage, contusion, etc. ....) (4)	☐	☐	☐	☐
Simple medical advice (guidance on what to do for a new health problem) (5)	☐	☐	☐	☐
Psychological support (6)	☐	☐	☐	☐
Request for a voucher for a consultation with another health professional (specialist, dietician, physiotherapist, etc.) (7)	☐	☐	☐	☐

Application or extension of work/sickness certificate (8)	☐	☐	☐	☐
Prescription renewal (9)	☐	☐	☐	☐

Q16 Since the COVID crisis, what changes in the ways you communicate with your patients have you experienced?

	More often (1)	Same thing (2)	Less often (3)	No use (4)
Telephone (1)	○	○	○	○
Mail (2)	○	○	○	○
Video (3)	○	○	○	○
Instant messaging (6)	○	○	○	○

Q17 What communication methods would you like to use for future consultations? (drag and drop)

______ Phone (1)
______ Mail (2)
______ Video (3)
______ Instant messaging (4)
______ Presential (5)

End of Block: Block 3

Start of Block: Block 4

Q18 In your opinion, what are/would be the barriers to the adoption of video consultation in your daily practice?

____

____

____

____

____

Q19 In your opinion, what are/would be the barriers to the adoption of telephone consultation in your daily practice?

____

____

____

____

____

Q20 What conditions do you think would encourage you to use telemedicine in your daily practice?

____

____

____

____

____

Q21 In your experience, what are the benefits of remote consultation with your patients? Please select up to 5 statements that you feel are most important from the following:

- ☐ This saves my patients a trip. (1)
- ☐ This saves my patients time (3)
- ☐ This makes it easier for my patients' family caregivers to participate. (6)
- ☐ This allows for a shorter consultation than in person. (7)
- ☐ This allows for a less expensive consultation than in person. (19)
- ☐ This saves my patients the trouble of arranging childcare. (8)
- ☐ This saves my patients from having to take time off work. (9)
- ☐ This allows my patients to get a quicker response or medical advice. (10)
- ☐ This allows for continued follow-up while traveling or living abroad. (11)
- ☐ This allows for closer medical follow-up than in person. (12)
- ☐ This saves my patients from going to an emergency room or another physician. (13)
- ☐ It allows my patients to talk about more things (15).
- ☐ This allows me to be more flexible on appointment times than in person. (16)
- ☐ This allows my patients to stay at home and feel less anxious about going to the doctor (less white coat effect). (17)
- ☐ ⊗I see no benefit to teleconsultation. (20)

Q23 In your opinion, what are the main disadvantages of communicating with your patients remotely? Please choose up to 5 statements that you feel are most important from the following:

- ☐ This does not guarantee confidentiality, as my patients are not always alone in their homes or workplaces. (1)
- ☐ This does not always guarantee data security depending on the platform used. (2)
- ☐ This exposes technical problems (equipment, connection, sound and/or image quality). (3)
- ☐ This sometimes requires downloading a specific software or application (4)
- ☐ This does not always lend itself to the medical situation. (5)
- ☐ This does not allow for close medical follow-up (6)
- ☐ This does not allow for a physical examination (7)
- ☐ This does not allow for good quality communication. (8)
- ☐ This does not allow my patients to ask all their questions because the time is limited. (9)
- ☐ This results in less participation from my patients. (10)
- ☐ This is associated with a less warm, less friendly contact. (11)
- ☐ This does not allow for physical contact (different from physical examination). (12)
- ☐ The timing of the teleconsultation is sometimes poor. (13)
- ☐ This requires special skills to communicate via video. (14)

☐ ⊗I see no disadvantage to teleconsultation. (15)

End of Block: Block 4

Start of Block: Block 5

Q22 Are you willing to be contacted by phone for an interview about telemedicine? If yes, please provide your name and phone number. (e.g. Mr. Muster 0221234567)

○ No (1)

○ Yes (2) ____

Q24 You have reached the end of the questionnaire. This space is reserved for you to share experiences or comments related to telemedicine.

____

____

____

____

____

End of Block: Block 5

Appendix 2 : Telemedicine- Parent Survey

Q1.1
You are invited to participate in a survey conducted by a team from the University Hospitals of Geneva (HUG) and the University of Geneva. It has been submitted to the Swiss Cantonal Commission for Research Ethics (exemption granted).

The aim of this study is to evaluate your perceptions and preferences concerning the modes of communication in telemedicine, i.e., for health care or advice given by telephone, video, email or instant messaging by your doctor. The results of this research will help to create recommendations for good practice.

Your participation consists of completing a questionnaire which should only take 10 minutes. You are free to accept or refuse to participate in the survey. You are able to withdraw from the project at any time without having to justify your decision.

The data collected for research purposes is anonymous. The analyzed results may be published in scientific journals. Confidentiality of the data will be ensured and your name or any identifying information will not be divulged. All participants involved in the study are protected by medical confidentiality.

You may contact the principal investigator of the study at any time for further information: telmed.hug@hcuge.ch

By agreeing to participate in this study, you agree to the use of your data for research purposes.

○ I wish to participate in this study. (1)

○ I do not wish to participate in this study. (2)

Q2.1 From which location are you filling out this survey?

- ○ HUG adult emergency department (1)
- ○ HUG pediatric emergency department (2)
- ○ Hôpital de La Tour-Meyrin adult emergency (3)
- ○ CPO-Onex adult emergency department (4)
- ○ CPO-Onex pediatric emergency department (5)
- ○ HUG Primary Care Medicine Service (6)
- ○ Vésenaz medical center (7)
- ○ Lancy medical center (8)
- ○ Hôpital de La Tour pediatric emergency department (11)
- ○ Other practice / medical center. (indicate below) (9)

____

- ○ Social networks (10)

Q3.1 In which year was your child born ? (eg 2007)

____

Q3.2 What is the gender of your child?

- ○ Boy (1)
- ○ Girl (2)
- ○ Personalized response (3) ____

Q3.3 Are you currently working?

- ◯ Yes, full time (1)
- ◯ Yes, part-time (2)
- ◯ No, I am not currently working (3)
- ◯ No, I am retired (4)
- ◯ ________

Q3.4 What is the highest level of education that you have completed?

- ◯ None (1)
- ◯ Compulsory school (2)
- ◯ Vocational training (3)
- ◯ Higher education (e.g. college, university) (4)
- ◯ Other (11) ____

Q4.1 Do you have internet access at home?

- ◯ Yes (1)
- ◯ No (2)
- ◯ I do not know (3)

Q4.2 Which devices do you own?

- ☐ Computer / laptop (1)
- ☐ Smartphone (2)
- ☐ Tablet (3)
- ☐ Other (4) ____
- ☐ ⊗None (5)

Q4.3 How often do you use these devices?

- ○ Everyday (1)
- ○ A few times per week (2)
- ○ A few times per month (3)
- ○ Less than once per month (4)
- ○ Never (5)

Q4.4 How do you use your devices?

- ☐ To make audio calls (1)
- ☐ To make video calls (2)
- ☐ To send and receive emails (3)
- ☐ To send and receive instant messages (e.g. SMS, WhatsApp) (4)
- ☐ To work (5)
- ☐ To play (6)

☐ To search for information (7)

Q5.1 During the last 12 months, how many times have you consulted one or more pediatrician for your child?

○ Once (1)

○ Twice (2)

○ 3-4 times (3)

○ 5-12 times (4)

○ More than 12 times (5)

Q5.2 Which of the following methods have you used to communicate remotely with a pediatrician?

☐ Telephone (1)

☐ Email (2)

☐ Video (3)

☐ SMS (or other instant messaging) (4)

☐ Other (5) ____

☐ ⊗None (6)

Q5.3 Do you have a pediatrician?

○ Yes (1)

○ No (2)

Q5.4 How long has he / she been your pediatrician?

- ◯ Less than 6 months (4)
- ◯ 6 months to 2 years (1)
- ◯ 2-5 years (2)
- ◯ More than 5 years (3)

Q6.1 Overall, do you think the health of your child is:

- ◯ Excellent (1)
- ◯ Very good (2)
- ◯ Good (3)
- ◯ Fair (4)
- ◯ Poor (5)

Q7.1 For each of the following situations, which means of communication with your pediatrician would be acceptable to you? (multiple choices possible)

	Telephone (1)	Email (2)	Video (3)	Instant messaging (4)
Receive exam results (labs, radiology) (1)	☐	☐	☐	☐
Discuss self-measured health data (weight, blood sugar level, pain, ...) (2)	☐	☐	☐	☐
Monitor a chronic health problem (nutrition, weight, behavior, ...) (3)	☐	☐	☐	☐
Follow up for a previously treated urgent problem (urinary tract infection, cold, gastroenteritis, contusion, etc.) (4)	☐	☐	☐	☐
Ask for simple medical advice (e.g. guidance on what to do for a new health problem) (5)	☐	☐	☐	☐
Receive psychological support (6)	☐	☐	☐	☐
Request for referal to another health professional (specialist doctor, dietician, physiotherapist, etc.) (7)	☐	☐	☐	☐
Request for a doctor's note / sick note (8)	☐	☐	☐	☐
Request for a prescription renewal (9)	☐	☐	☐	☐

Q7.2 If you had to choose, which means of communication would you prefer for each of the following situations? (only one choice possible):

	Telephone (1)	Email (2)	Video (3)	Instant messaging (4)
Receive exam results (labs, radiology) (1)	○	○	○	○
Discuss self-measured health data (weight, blood sugar level, pain, ...) (2)	○	○	○	○
Monitor a chronic health problem (nutrition, weight, behavior, ...) (3)	○	○	○	○
Follow up for a previously treated urgent problem (urinary tract infection, cold, gastroenteritis, contusion, etc.) (4)	○	○	○	○
Ask for simple medical advice (e.g. guidance on what to do for a new health problem) (5)	○	○	○	○
Receive psychological support (6)	○	○	○	○
Request for referal to another health professional (specialist doctor, dietician, physiotherapist, etc.) (7)	○	○	○	○
Request for dosctor's note / sick note (8)	○	○	○	○
Request for a prescription renewal (9)	○	○	○	○

Q7.3 Since the beginning of the COVID crisis, what changes have you experienced regarding how you communicate with your pediatrician?

	More often (1)	No change (2)	Less often (3)	Not applicable (4)
Telephone (1)	○	○	○	○
Email (2)	○	○	○	○
Video (3)	○	○	○	○
Instant messaging (4)	○	○	○	○

Q7.4 Rank the following means of communication in order of preference for your future consultations: (drag and drop)

______ Telephone (1)
______ Email (2)
______ Video (3)
______ Instant messaging (4)
______ Face-to-face (5)

Q8.1 In your case, what are the main advantages of remote consultation (by phone or video) with a pediatrician? (5 maximum)

- [ ] It saves me a trip. (1)
- [ ] It saves me time. (3)
- [ ] It makes it easier for my family caregivers to participate. (5)
- [ ] It allows for a shorter consultation than in person (6)
- [ ] It allows for a less expensive consultation than in person (18)
- [ ] It avoids having to arrange childcare (7)
- [ ] It avoids having to take time off work. (8)
- [ ] I have faster access to medical care or advice. (9)
- [ ] I can continue (maintain) follow-up while traveling abroad. (10)
- [ ] It allows me to have more frequent follow-up than in person. (11)
- [ ] It avoids having to visit an emergency center or a different pediatrician. (12)
- [ ] It allows me to talk about more things than in person. (14)
- [ ] It allows the doctor to be more punctual about the appointment time than in person. (15)
- [ ] Staying at home allows me to feel less anxious than in coming in person (16)
- [ ] ⊗ I do not see any benefit to teleconsultations. (20)

Q8.2 In your opinion, what are the main disadvantages of remote communication with a doctor? (maximum 5 choices)

- ☐ It does not guarantee confidentiality of the consultation (I am afraid of being overheard). (1)
- ☐ It does not guarantee data security (the means used may not be secure). (2)
- ☐ Technical problems may arrive (equipment, connection, and sound and/or image quality). (3)
- ☐ It requires downloading a specific software or application. (4)
- ☐ It does not always lend itself to the medical situation. (5)
- ☐ It does not allow for frequent health monitoring. (6)
- ☐ It does not allow for a physical examination. (7)
- ☐ It does not allow for high quality communication. (8)
- ☐ We talk about fewer things than in person. (9)
- ☐ It does not allow me to participate as actively as in person. (10)
- ☐ It offers a less warm, less friendly contact than in person. (11)
- ☐ It does not allow for physical contact (12)
- ☐ It gives me the impression that the doctor is intruding on my privacy during a video consultation. (13)
- ☐ The call can come at a bad time. (14)
- ☐ ⊗ I don't see any disadvantages to teleconsultation. (15)

Q9.1 Indicate your level of agreement with the statements below.

	Strongly agree (1)	Agree (2)	No opinion (3)	Disagree (4)	Strongly disagree (6)
I am confident that video communications are secure and confidential. (1)	○	○	○	○	○
I am confident that telephone communications are secure and confidential. (2)	○	○	○	○	○
I can communicate everthing I need to in a video consultation. (5)	○	○	○	○	○
I can communicate everthing I need to in a telephone consultation. (6)	○	○	○	○	○
The video consultation allows the pediatrician to understand my child's health problem. (7)	○	○	○	○	○
The telephone consultation allows the pediatrician to understand my child's health problem. (8)	○	○	○	○	○
I can expect the same quality of care via video as I would in person. (9)	○	○	○	○	○
I can expect the same quality of care via telephone as I would in person. (10)	○	○	○	○	○

Q10.1 Did you complete this questionnaire with your own support using the QR code?

- ◯ Yes (1)
- ◯ No (2)

Q10.2 Please enter your email in order to receive the compensation for your time and effort.

____

Q10.3 Are you willing to be contacted by phone for an interview about telemedicine? If so, please provide your name and phone number in the space below. (e.g. Mr. Muster 022 4567809)

- ◯ No (1)
- ◯ Yes (2) ____

Q10.4 Vous êtes arrivé à la fin de ce questionnaire. Cet espace est à votre disposition pour vos remarques ou expériences personnelles en lien avec la téléconsultation.

____

____

____

____

____

Q10.4 You have reached the end of this questionnaire. This space is at your disposal for your comments or personal experiences related to teleconsultations.

____

____

____

____
